# Impact of different water activities (*a*_w_) adjusted by solutes on high pressure high temperature inactivation of *Bacillus amyloliquefaciens* spores

**DOI:** 10.3389/fmicb.2015.00689

**Published:** 2015-07-06

**Authors:** Robert Sevenich, Kai Reineke, Philipp Hecht, Antje Fröhling, Cornelia Rauh, Oliver Schlüter, Dietrich Knorr

**Affiliations:** ^1^Department of Food Biotechnology and Food Process Engineering, Technische Universität Berlin, BerlinGermany; ^2^Leibniz Institute for Agriculture Engineering (ATB), PotsdamGermany

**Keywords:** High pressure high temperature, reduced water activity, baroprotective effect, *Bacillus amyloliquefaciens*, spore inactivation

## Abstract

Much research has been conducted to comprehend the mechanisms of high pressure (HP) inactivation of spores in aqueous systems but for food model systems these information are scarce. In these systems spores can interact with ingredients which then could possibly lead to retarded or reduced inactivation, which can cause a problem for the sterilization process. The protective mechanism of a reduced *a*_w_-value is still unclear. HP processing might prove valuable to overcome protective effects of solutes and achieve shorter process times for sterilization under HP. To gain insight into the underlying mechanisms five *a*_w_-values (0.9, 0.92, 0.94, 0.96, 1) were adjusted with two different solutes (NaCl, sucrose). Solutions were inoculated with spores of *Bacillus amyloliquefaciens* and treated at 105, 110, and 115°C at 600 MPa. Further a thermal inactivation was conducted at the same temperatures for a comparison with the HP data. Afterward, the influence of HP high temperature treatment on the inactivation, the dipicolinic acid (DPA)-release and membrane constitution was assessed by plate count, HPLC and flow cytometry (FCM). The results show that during HP treatments sucrose and salt both have a protective effect, in which the influence of sucrose on the retarded inactivation is higher. The threshold water activities (*a*_w)_, which is 0.94, here salt and sucrose have a significant influence on the inactivation. The comparison of thermal (105–115°C) and HP and high temperature (600 MPa, 105–115°C) treated samples showed that the time needed to achieve a 4–5 log_10_ inactivation is reduced from 45 (*a*_w_ = 1) to 75 (*a*_w_ = 0.9) min at 105°C to 3 (*a*_w_ = 1) to 15 (*a*_w_ = 0.9) minutes at 600 MPa and 105°C. The release of DPA is the rate limiting step of the inactivation and therefore monitoring the release is of great interest. The DPA-release is slowed down in high concentrated solutions (e.g., sucrose, salt) in comparison to *a*_w_ 1. Since there is a difference in the way the solutes protect the spore it could be seen as an inner spore membrane effect. Maybe as shown for vegetative microorganism the solutes can interact with membranes, e.g., the inner spore membrane. Flow cytometry (FCM) measurement data show a similar trend.

## Introduction

One of the main aims of the food industry is the product safety but in the recent years due to consumer demand the product quality of the food has also gained more importance (Ramirez et al., 2009; Olsen et al., 2010; Belletti et al., 2013; Reineke et al., 2013a). The combination of elevated temperatures (90–121°C) and high pressure (HP) processing (up to 600 MPa) show a high technological potential to preserve foods in a more gentle and efficient way then only by heat processes. In the recent years a lot of research has been conducted to understand the influence of the so called high pressure thermal sterilization (HPTS) and its impact on; spores, food processing contaminants, the working principle of the technology and synergism of temperature and pressure (Margosch et al., 2006; Reineke et al., 2013a; Georget et al., 2015; Olivier et al., 2015; Sevenich et al., 2015). The mechanisms of spore inactivation under these servere conditions will not be discussed in detail since they are well described in literature elsewhere (Wuytack et al., 1998; Setlow, 2003; Margosch et al., 2006; Black et al., 2007; Barbosa-Canovas and Juliano, 2008; Reineke et al., 2012, 2013b). Although, it should be mentioned, since it is of importance for this work, that the release of dipicolinic acid (DPA), which makes up 5–15 % of the dry matter content of the spores, is thought to be the rate limiting step of the inactivation (Reineke et al., 2013b). At pressures above or equal 400 MPa an opening of the Ca^2+^ – DPA – channels occurs: (i) DPA is released from the spore core; (ii) the spore core gets dehydrated; and (iii) therefore, it becomes thermo- and pressure sensitive and can be inactivated (Reineke et al., 2012). If the treatment temperature is moderate (≤50°C) the spore will be triggered to germinate. For a rapid and sudden inactivation of spores under pressure it is important to apply pressures ≥600 MPa and temperatures above 60°C to ensure the loss of heat resistance (Reineke et al., 2013b).

One possible explanation of the non-implementation of HPTS could be the fact that certain ingredients of foods such as fats, sugars, salts and the resultant water activities could lead to a retarded or reduced inactivation under these conditions. The so called baroprotective effect is not well studied yet for spores in combination with HP and high temperatures. Water activity (*a*_w_) is a major factor in preventing or limiting growth (bacteria causing food borne diseases will not grow under *a*_w_ of 0.85) but further it can also lead to an increased resistance of microorganisms and spores. The influence of salts or sugars on the *a*_w_ of foods or suspension media does not explain the marked baroprotective effects of these solutes and it has been suggested that specific interactions between solutes and biological macromolecules contribute to their baroprotective effects (Fujii et al., 1996; Molina-Höppner et al., 2004). These observations were already made by researchers in the 1950–1980, which tried to understand the protective effect of solutes (sodium chloride, sucrose, glucose, fats, acids etc.) and the precipitated change in *a*_w_ during the thermal inactivation of microorganisms (Anderson et al., 1949; Secrist and Stumbo, 1958; Murrell and Scott, 1966; Molin and Snygg, 1967; Cook and Gilbert, 1968; Beuchat, 1974; Gould, 1977; Härnulv et al., 1977; Senhaji and Loncin, 1977). These researchers indicated that a protective effect of solutes/*a*_w_-value exists and can lead to an increase of the *D*-value for the tested microorganisms. Some further stated that the protective effect is depending on the concentration of solute, the solute itself (ionic e.g., salt or non-ionic e.g., sugar), the selected microorganism and the temperature. However, the protective mechanism of a reduced *a*_w_-value is still unclear. Molin and Snygg (1967) reported in their studies that fats/oils increased the heat resistance of *Bacillus cereus* and *B. subtilis* spores. They stated that the protective effect is not only due to the low heat conductivity or the water free environment. A more apparent possibility for that protective effect might be that the free fatty acids present in some oils could have a stabilizing effect on spores.

The use of an emerging technology such as HP processing might prove valuable to overcome the protective effect of the solutes. And by doing so, achieve shorter process times. Some interesting studies concerning the influence of HP on baroprotecive solutes exist. Oxen and Knorr (1993) showed that HP inactivation of *Rhodotorula rubra* in different *a*_w_ solutions was more effective than by heat alone. Molina-Höppner et al. (2004) stated that microorganisms (*Lactococcus lactis, Escherichia coli*) due to the osmotic up-shock accumulate solutes (NaCl and sucrose) which then can interact with biomolecules, as per the example of phospoholipid bilayers. Sucrose preserved the metabolic activity and membrane integrity of the cells during the high-pressure treatment, whereas salt preserved the membrane integrity but not the metabolic activity. Due to the accumulation, the membrane stays in a more fluid state during the treatment and therefore shows a higher HP resistance. However, the baroprotection of salt (ionic solutes) requires higher concentrations than the same baroportection by disaccharides.

No such detailed research was conducted at this point for spores but it is plausible that also spores could accumulate solutes by diffusion in the inner compartments, e.g., inner spore membrane, where these could interact with biomolecules. Some prior works already indicated that such a phenomenon could exist in food systems (cacao mass, fish systems, baby food puree, food concentrates) for HP high temperature processes as well (Ananta et al., 2001; Sevenich et al., 2013, 2014, 2015; Georget et al., 2015). Gerhardt and Black (1961) and Black and Gerhardt (1962) stated, that the central core of a spore is kept relatively dry but that the rest of the compartments are free to extensive water and solute permeation. Other authors mentioned that the predominant compartment that plays an important role in the uptake of solutes is the spore cortex. The cortex possesses a negative net charge. The low degree of cross-linking supposes that the spore cortex is able to change volume in response to changes as a result of balancing the electrostatic interaction (Kazakov, 2008; Kazakov et al., 2008). These induced changes in the different spore compartments could have an impact on the inactivation mechanism under HP. Another possible postulated impact of the baroprotective of solutes could be the influence on the rate limiting step of the spore inactivation under pressure, which is the release of DPA out of the spore core and the followed rehydration of the core by water. Lower *a*_w_ means that most of the outside water is bound to solutes and cannot freely move. Therefore, one could assume that the exchange of water and DPA is slowed down due to changes in the osmotic pressure or the solubility of DPA in high concentrated solutions might differ, which both could result in retarded and delayed inactivation. In addition, a third postulated impact could be that high concentrations of solutes close to the oversaturation point can inhibit the pressure transmission of water to some degree and therefore generate an insufficient and in-homogenous pressure distribution within the system (Eder and Delgado, 2007; Min et al., 2010). The spores might, if the threshold pressure (500–600 MPa) is not reached, not undergo the postulated pathways of germination/inactivation.

The aim of this investigation was to obtain insights into mechanisms of baroprotection by NaCl and sucrose on *B. amyloliquefaciens* spores (TMW FAD82), which are very HP high temperature resistant (Margosch et al., 2006; Sevenich et al., 2013), under HP thermal sterilization conditions (600 MPa; 105, 110, and 115°C). Therefore, solutions with different *a*_w_ were prepared with two different solutes one was sodium chloride (1.2–2.7 mol/L) and the other sucrose (0.83–1.7 mol/L). The influence of the two different solutes and their resultant *a*_w_ values on inactivation was monitored by plate count, the DPA-release by HPLC and changes in the membrane barrier properties by flow cytometry (FCM).

## Materials and Methods

### Sporulation, Spore Preparation, and *a*_w_-Solution Preparation

Using a method described elsewhere (Paidhungat et al., 2002), sporulation of *B. amyloliquefaciens* (Technische Mikrobiologie Weihenstephan, 2.479, Fad 82) was induced at 37°C on solid 2 × SG medium agar plates without antibiotics. The harvest was carried out when 90% of the spores were phase bright under the light microscope, which took 2–3 days. The spore suspension was cleaned by repeated centrifugation (threefold at 5000 *g*), washed with cold distilled water (4°C), and was treated with sonication for 1 min. The cleaned spore suspensions contained ≥95% phase bright spores and nearly no spore agglomerates, as was verified by a particle analysis system (FPIA 3000, Malvern Instruments, Worcestershire, UK). The spore suspensions were stored in the dark at 4°C.

Five different *a*_w_-values were used for each solute (NaCl and sucrose); the range was selected from 1 to 0.9 (1, 0.96, 0.94, 0.92, 0.90), since this represents the *a*_w_ of many relevant food systems. To adjust the *a*_w_ with NaCl and sucrose a table published by the Food Safety Bulletin was used (Food-Saftey, 2010). The amounts of water (aqua dest.) and solute for the corresponding *a*_w_ were mixed in 500 ml flasks (Schott Ag, Mainz, Germany) and afterward autoclaved to gain a homogenous solution. After cooling down, the flasks were stored at 4°C.

### High Pressure High Temperature Treatment

Twenty-four hours prior to the each HP trial *B. amyloliquefaciens* spores were suspended into the *a*_w_-value solution to give the spores time to adapt to the new surroundings. The total cell count of the solution was between 10^7^ and 10^8^spores/ml. From these spore suspension 3^∗^300 μl (one for the inactivation kinetics, one for DPA analyses and one for FCM analyses) were filled in shrinking tubes (Schrumpfschlauch 3/1, DSG-Canusa, Meckenheim, Germany, inner diameter 3 mm, outer diameter 3.6 mm) and were hermitically sealed with a soldering iron. All three shrinking tubes were put in 2 ml containers (2 mL, CryoTube Vials, Nunc Brand Products, Roskilde, Denmark) filled with same solution. For the pressure trials the U111 Monovessel unit (Unipress, Warsaw, Poland), with a 3.7 mL vessel volume and a compression rate of 25 MPa/s was used. In this pressure equipment, Di-2-ethyl-hexyl-sebacate served as the pressure-transmitting medium. To reach the designated treatment temperature, the pressure vessel was immersed in a thermostatic bath (cc2, Huber GmbH) filled with silicon oil (M40.165.10, Huber GmbH). To monitor the temperature during the treatment the temperature was measured in the geometrical center of container by a thermocouple. The temperatures selected for the treatment were 105, 110, and 115°C at 600 MPa with isothermal dwell times between 0.0166 and 15 min. The oil bath was set on the selected process temperature and the start temperatures for each food system were obtained in a pretrial. Before and after the trials the samples were stored on ice. After the treatments one of the shrinking tubes was used for the appropriate dilutions of each sample for surface-plated on nutrient agar (CM 003, Oxoid Ltd., Hampshire, England). The dishes were incubated at 37°C for 2 days, and the colony-forming units (CFUs) were counted. All trials were conducted in duplicates. The samples for the DPA or FCM analyses were put in 1 ml reaction cups (1 mL, CryoTube Vials, Nunc Brand Products, Roskilde, Denmark) and stored at -80°C until further analysis.

### Thermal Inactivation of Spores Suspended in the Different Water Activities

The thermal sensitivity of *B. amyloliquefaciens* spores (10^7^ CFU/ml) in the different *a*_w_-values was conducted at ambient pressure in the temperature range of 105–115°C. Static temperature treatments of the spores were performed in a temperature-controlled oil bath using glass capillaries (Hirschmann Labogeräte, inner diameter 1 mm and outer diameter 1.3 mm, Germany). This was done to ensure instantaneous heating and cooling of the solutions. After the treatment, samples were immediately transferred to an ice bath to prevent further inactivation. The samples were diluted and incubated as described under section “High Pressure High Temperature Treatment.”

### DPA-Analyses by HPLC

Prior to the DPA analyses the samples were filtered through 0.2 μm Nylon filters (Rotilabo^®^ Spritzenfilter; Carl Roth GmbH & Co KG, D-76185 Karlsruhe) to remove spores and other particles which could interfere with the analyses. A Dionex Ultimate 3000 system was used (Dionex Corporation, Sunnyvale, CA, USA), with a reversed phase separating column (RP 18—5 μm LiChroCART 124-4; Merck, KGaA, Darmstadt, DE) that was protected with a guard column (LichroCART 4-4; Merck KGaA, Darmstadt, DE). The DPA detection limit of this HPLC setup was 1 μM. To determine the total amount of DPA in the spore suspensions, 1 mL of each individual batch was thermally treated at 121°C for 20 min (Reineke et al., 2013b) and then analyzed. To identify the peaks and to determine the DPA amount released standard solutions of known DPA concentrations were used to calculate a calibration curve. Pressure-induced DPA release was calculated relative to the total DPA content of each individual spore batch, and all of the data are represented as the mean of at least two independent experiments.

### Flow Cytometry

For the FCM analyses a method described by Mathys et al. (2007) was used. For the sample preparation a double staining was used, involving SYTO16 (Invitrogen, Carlsbad, CA, USA) and propidium iodide (PI; Invitrogen, Carlsbad, CA, USA). Both fluorescent dyes are able to stain DNA, but the membrane permeant SYTO16 acts as an indicator for spore germination, whereas the membrane impermeant PI indicates membrane damage. The treated spore suspensions were diluted with *N*-(2-Acetamido)-2-aminoethanesulfonic acid (ACES) buffer solution (0.05 M, pH 7) to achieve a flow rate of about 1000 events/s, as well as a constant ratio of spores for the staining. The concentrations of the fluorescent dyes for staining were 15 μM PI and 0.5 μM SYTO16 in the diluted spore suspension. Afterward, the samples were stored in the dark at ambient temperature for 15 min. The analyses were carried out using a CyFlow ML flow cytometer (Sysmex Partec, GmbH, Münster, Germany) equipped with Partec FlowMax Operating and Analysis Software for Partec Flow Cytometry Particle Analyzing systems Version 3.0 (b5) (January 12 2009) was used as operation and acquisition software. Excitation was set to 488 nm and the fluorescence of Syto 16 was measured with the photomultiplier FL1 and a band pass filter with 536 ± 20 nm cut off. For the fluorescent of PI the photomultiplier FL3 and a short pass filter with 620 nm cut off ±15 nm (fluorescence intensity is recorded between 615 and 620 nm), was used. The parameters were collected as logarithmic signals and the obtained data was analyzed using Software FCS Express Version 4 for Flow and Image Cytometry Analysis (De Novo Software, Los Angeles, CA, USA). All analyses were performed in triplicates. Only certain samples (a minimum, intermediate, maximum *a*_w_ (1, 0.94, 0.90) for sucrose and NaCl in a temperature range of 105–115°C) were analyzed to get an overview of the influence of solutes on the physiological state of spores.

### Calculation of Isokineticity Lines

For the calculation of the isokinetic lines the Weibullian approach (Equation 1) was used. This approach is a vitalistic approach and is suitable to describe inactivation kinetics of HP processing (van Boekel, 2002; Juliano et al., 2009; Reineke et al., 2013a).

(1) $$\text{log}\left(\frac{N}{N_{0}}\right)\;=\;-{\left(\frac{t}{\Delta }\right)}^{b}$$

With Δ as the scale parameter, b as the shape parameter. The shape parameter, b, determines the curve progression of the inactivation curve, and simultaneously gives information about the dying behavior of the respective microorganisms. For values of b smaller than 1 the decreasing curve becomes increasingly flatter. This implies that the microorganisms, not yet killed, are more resistant to the treatment than the microorganisms that have already been killed. If b equals 1 the graph is linear and therefore corresponds to a first order inactivation. For values of b greater than 1 the inactivation curve decreases progressively it can be deduced that the remaining cells exhibit increasing sensitivity against the applied treatment (van Boekel, 2002).

The parameter b and Δ were obtained by using the Weibull fit of the analytical software “Geeraerd and Van Impe Inactivation model Fitting Tool” (GinaFit Version 1.6 March 2012, Katholieke Universiteit Leuven). The mean shape parameter b for all inactivation kinetics was calculated and all inactivation-kinetics were refitted with the mean b to obtain Δ. To get a functional dependency of Δ (T), Δ and T were fitted with all equation set of TableCurve3D (SPSS Inc., Chicago, IL, USA). The equation with the minimal sum of square errors for Δ (T) was then used in (1) for Δ. The isorate lines were calculated with MathCAD 2001i professional (Mathsoft Engineering & Education, Inc., USA).

Statistical analyses: the statistical analysis of the data was performed using Statgraphics (Version 4.0, StatPoint Technologies, Warrenton VA, USA) Multiple range test was used to analyze the significance of the tested data. Significance for all statistical analysis was defined as *p* < 0.05.

## Results

### High Pressure High Temperature in Comparison to Thermal only Inactivation of *Bacillus amyloliquefaciens* in Different Water Activities

**FIGURE 1:**
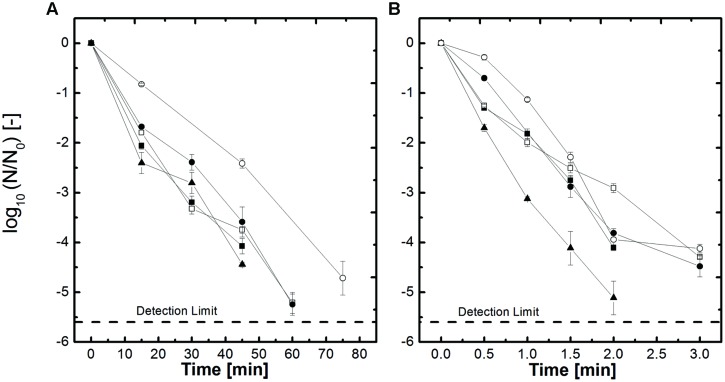
**Effect of pressure and heat on the inactivation of *Bacillus amyloliquefaciens* spores in selected water activities adjusted by NaCl and sucrose **(A)** 600 MPa, 105°C and **(B)** 600 MPa, 115°C.** *a*_w_ = 1 (▲), *a*_w_ = 0.94 NaCl (■), *a*_w_ = 0.90 NaCl (□), *a*_w_ = 0.94 sucrose (●) and *a*_w_ = 0.90 sucrose (○). Initial spore count ~10^7^ CFU/ml.

Spores of *B. amyloliquefaciens* inoculated in different *a*_w_, adjusted by NaCl and sucrose, were used to investigate the influence of the baroprotective effect of these solutes on the HP high temperature and thermal only inactivation. In **Figures 1A,B** selected inactivation kinetics of the tested kinetics are shown to give an overview of the inactivation behavior (105°C 600 MPa and 115°C 600 MPa). **Figure 1A** shows the inactivation kinetics at 600 MPa, 105°C for the *a*_w_-values 1, 0.94, and 0.90 for NaCl and sucrose. It becomes obvious that the higher the concentration of the solute, respectively the lower the *a*_w_, the slower the inactivation becomes. Sugar has a more severe impact on the retardation of the inactivation as NaCl. If one looks at the inactivation kinetics *a*_w_ = 0.9 of NaCl it takes 8 min to achieve a 4.2 log_10_ inactivation whereas a similar inactivation for sucrose with the same *a*_w_ takes 15 min. Interesting to see is that an *a*_w_ = 1 and *a*_w_ = 0.94 adjusted with salt shows equal inactivation behavior. This underlines that not only the *a*_w_ needs to be taken into account but the solute as well. The other inactivation data which are not shown show similar trends. If the temperature is increased by 10°C up to 115°C (**Figure 1B**) the treatment times in comparison to 105°C, 600 MPa (**Figure 1A**) to achieve inactivation of 4–5 log_10_ are much shorter. Further the graphs converge closer together, which can be seen as an indication that the applied temperature is able to overcome the protective effect.

**FIGURE 2:**
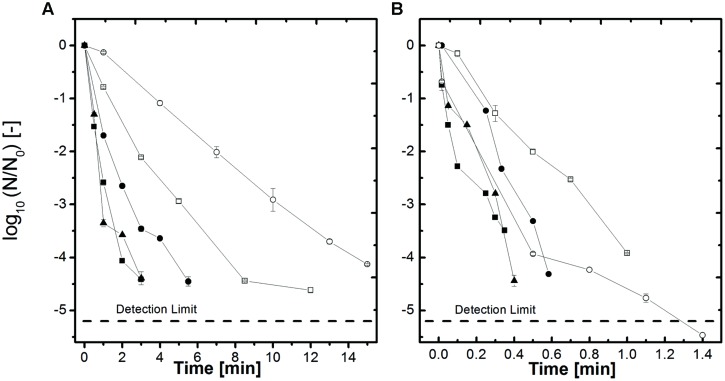
**Effect of heat on the inactivation of *B. amyloliquefaciens* spores in selected water activities adjusted by NaCl and sucrose **(A)** 105°C and **(B)** 115°C.** *a*_w_ = 1 (▲), *a*_w_ = 0.94 NaCl (■), *a*_w_ = 0.90 NaCl (□), *a*_w_ = 0.94 sucrose (●) and *a*_w_ = 0.90 sucrose (○). Initial spore count ~10^6^ CFU/ml.

The inactivation results for the thermal treatment at 105–115°C of *B. amyloliquefaciens* spores in the different *a*_w_-solutions (here only *a*_w_ = 1, 0.94, and 0.90 are shown) adjusted by NaCl and sucrose are depicted in **Figure 2**. In comparison to the HP treated samples the time needed to achieve a 4 log inactivation at 105°C only is between 45 and 65 min (**Figure 2A**) whereas at 600 MPa. 105°C (**Figure 1A**) the dwell time is between 2 and 14 min. At 115°C (**Figure 2B**) the inactivation rates are higher but still the HP treated samples at the same temperature (**Figure 1B**) are inactivated slightly quicker. The behavior of the spores in the different *a*_w_-solutions for the thermal only inactivation is the same as described for the HP treated samples.

**FIGURE 3:**
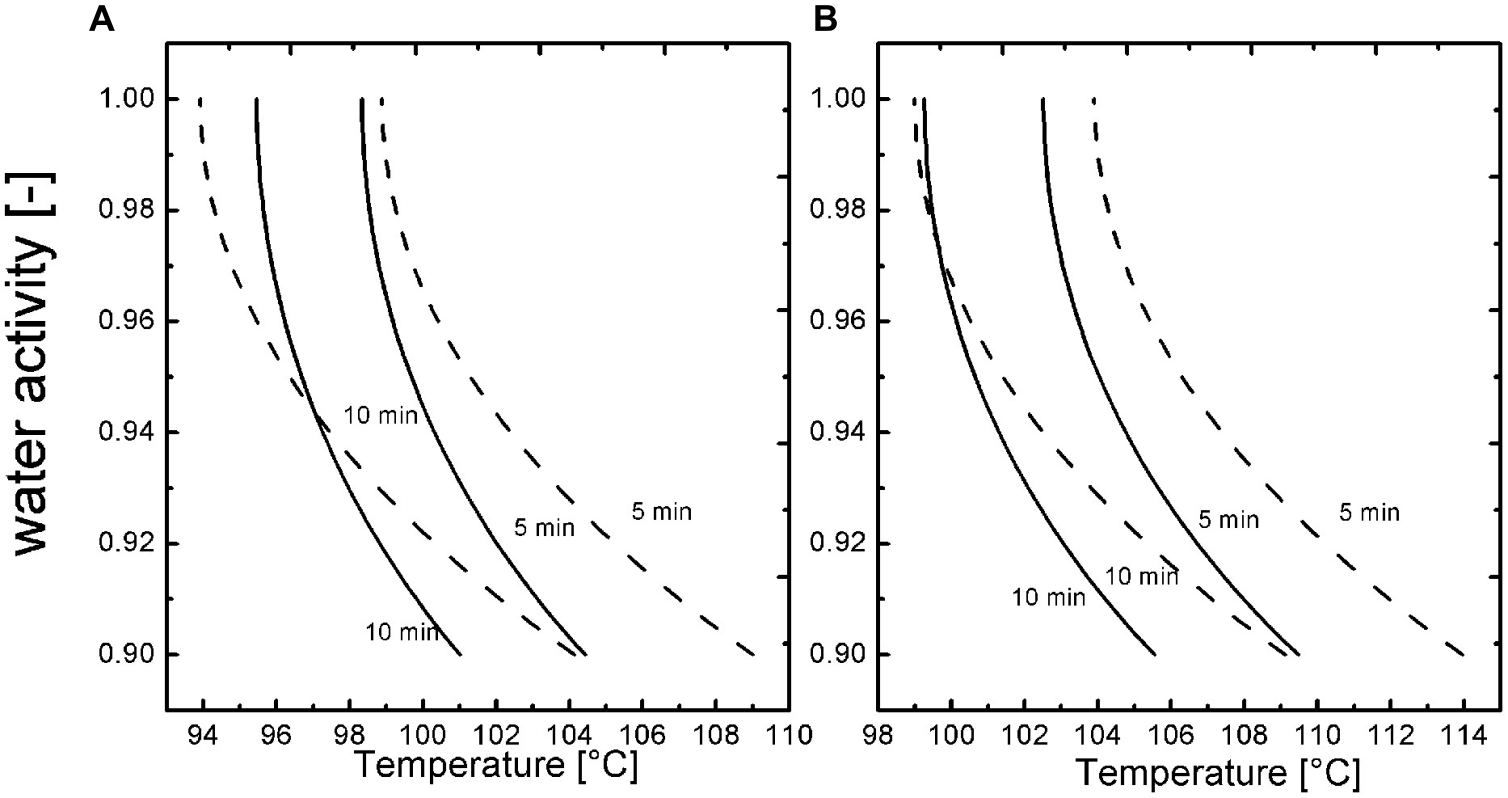
**Isokientic lines of weibull approach for NaCl (solid line; *b* = 0.57) and sucrose (dashed line *b* = 0.72) **(A)** -3 log_10_ and **(B)** -5 log_10_ inactivation at 600 MPa**.

To gain a better understanding of the T, t dependencies at 600 MPa a modeling for the spore inactivation in the different *a*_w_ (1, 0.96, 0.94, 0.92, 0.90) adjusted with NaCl and sucrose was conducted based on the obtained inactivation kinetics for a 3 log_10_ and 5 log_10_ inactivation of *B. amyloliquefaciens* (**Figures 3A,B**). The inactivation that was achieved during the pressure build up (kinetic point of 1 s) was subtracted from the other kinetic points of each temperature, to have a valid model for isothermal and isobaric conditions. **Figure 3** shows the most relevant domain of *a*_w_ for the food industry and the influence on the inactivation of spores depending on the solute. A 3 and 5 log_10_ inactivation of *B. amyloliquefaciens* is possible for all tested *a*_w_ and solutes in a time range of 5–10 min and temperatures between 94 and 114°C at 600 MPa. The isokinetic lines also depict that sucrose has a more pronounced baroprotective effect then NaCl and therefore higher temperatures are needed to achieve the same kind of inactivation.

### Quantitative Analyses of DPA-Release in the Different Water Activities by HPLC

Release of DPA during the HP-induced spore inactivation was monitored by HPLC to draw inferences from the released DPA about the physiological state of the cells, respectively, the inactivation in the presence of solutes (sucrose and NaCl in *a*_w_ range 0.90–1). Here, the ability of the spores to retain DPA can be seen as an indicator of the pressure and heat resistance of spores (Margosch et al., 2004). Furthermore, Reineke et al. (2013b) found that the release of DPA appears to be the rate-determining step of spore inactivation for a combined pressure and heat treatment. To determine the relative DPA-release in the tested spore population the maximum amount of available DPA was quantified by batch sterilization in an autoclave for 20 min at 121°C. The measured amount of released DPA was used as the maximum detectable DPA content and was set in relation to the treated samples. For the released DPA only the endpoints of the corresponding treatment and *a*_w_ were used. An overview of the relative DPA-release and the corresponding treatment time for the different *a*_w_ is given for a temperature range of 105–115°C in **Tables 1** and **2**. In general one can state for the *a*_w_ adjusted with NaCl and sucrose (**Tables 1** and **2**) that the DPA-release is depending on the *a*_w_ (≤0.94). For NaCl the analyses showed that with increasing temperature the time to release the same amount of DPA is getting shorter and the released amounts increase to 84–92 % at 115°C in comparison to 72–92% at 105°C (**Table 1**). For higher temperatures the protective effect diminishes and is only more or less present for the *a*_w_ 0.92 and 0.90. In comparison to NaCl, the DPA release into the sucrose solutions (**Table 2**) shows the same tendencies described for NaCl. However, the temperature increase to 110°C, 600 MPa (**Table 2**) does not have such a huge impact on the DPA release and time; as for NaCl (**Table 1**). At 115°C, 600 MPa (**Table 2**) the impact of temperature becomes dominant but not as intense as for NaCl.

**Table 1:** Relative DPA-release in % at maximal treatment time and corresponding temperature of *Bacillus amyloliquefaciens* in different water activities adjusted by NaCl.

	Water activity [-]	Max. DPA-release [%]	SD Maximum DPA-release [%]	Maximum treatment time [min]
NaCl, 105°C, 600 MPa	1	82.3	0.276	3
	0.96	92.64	2.75	2
	0.94	84.84	2.17	3
	0.92	81.23	0.196	5
	0.9	72.76	2.58	12

NaCl, 110°C, 600 MPa	1	95.15	0.245	1
	0.96	94.39	1.352	2
	0.94	89.06	0.44	3.5
	0.92	90.53	0.801	3
	0.9	89.81	0.129	3

NaCl, 115°C, 600 MPa	1	92.65	3.26	0.3
	0.96	91.44	0.832	0.5
	0.94	85.53	5.56	0.3
	0.92	87.66	1.87	0.7
	0.9	84.1	0.461	1

**Table 2:** Relative DPA-release in % at maximal treatment time and corresponding temperature of *B. amyloliquefaciens* in different water activities adjusted by sucrose.

	Water activity [-]	Maximum DPA-release [%]	SD Maximum DPA-release [%]	Maximum treatment time [min]
Sucrose, 105°C, 600 MPa	1	82.3	0.276	3
	0.96	82.82	0.538	4
	0.94	84.61	0.454	5.5
	0.92	89.22	0.186	13
	0.9	49.71	0.7588	15

Sucrose, 110°C, 600 MPa	1	95.15	0.245	3
	0.96	89	0.448	2.5
	0.94	87	3.52	3.5
	0.92	85.54	2.69	6
	0.9	86.36	0.767	11

Sucrose, 115°C, 600 MPa	1	92.65	3.26	0.3
	0.96	85	2.49	0.25
	0.94	86	2.33	0.85
	0.92	88.57	4.18	1.25
	0.9	88.19	2.21	2

### Flow Cytometry Analyses to Identify Possible Changes in the Membrane Constitutions by Solutes

**FIGURE 4:**
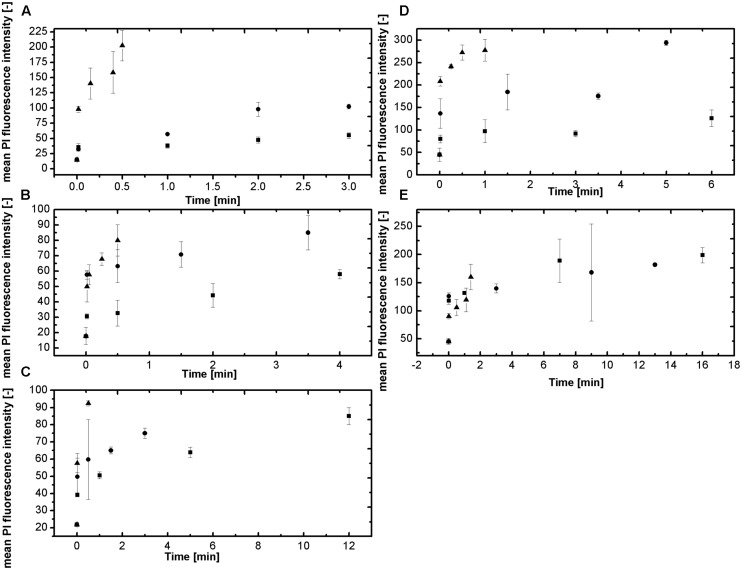
**Mean PI fluorescence intensity for the tested water activities at 600 MPa and a temperature range of 105–115°C (■ 105°C, ● 110°C, and ▲ 115°C). (A)** *a*_w_ = 1; **(B)** 0.94 NaCl; **(C)** *a*_w_ = 0.90 NaCl; **(D)** *a*_w_ = 0.94 sucrose and **(E)** *a*_w_ = 0.90 sucrose.

The method uses a double staining approach, the membrane permeable Syto16, is as an indicator for germination, since staining with this dye is not possible until the degradation of the spore cortex was initiated (Black et al., 2005). The membrane impermeable PI, is an indicator for the spore inactivation since the rupture of the inner spore membrane is necessary for its detection. For both fluorescent colorants there have been no documented interactions with sucrose or NaCl. This at least can be disapproved by the following results. The staining of the spores in the different *a*_w_ adjusted by solutes was quite difficult. Since for higher solute concentrations the viscose and concentrated solutions seemed to keep the dye away from the spores and staining might be insufficient. This is depicted in **Figure 4** where the mean PI fluorescence intensity is shown over the treatment time and temperature for the corresponding *a*_w_. The mean PI fluorescence intensity describes the average intensity of PI of the spores over the entire set of detection channels and therefore can be used as an indicator for the influence of HP high temperature on the inner spore membrane. The PI concentration increases with increasing temperature but decreases with *a*_w_. For **Figures 4A–C** this trend is obvious and indicates the influence of NaCl on the inner spore membrane with increasing *a*_w_-value. The interpretation of the results of sucrose (**Figures 4D,E**) is quite difficult. The influence of the solute, as described for NaCl, cannot be verified by the obtained results. Tendencies are present for *a*_w_ 0.94 (**Figure 4D**) but *a*_w_ 0.9 does not follow a clear trend. Although, the results of the DPA-release and the inactivation in sucrose solutions seemed to indicate this kind of influence. Maybe the solute concentration/dye ratio has an influence on the staining properties of PI.

**FIGURE 5:**
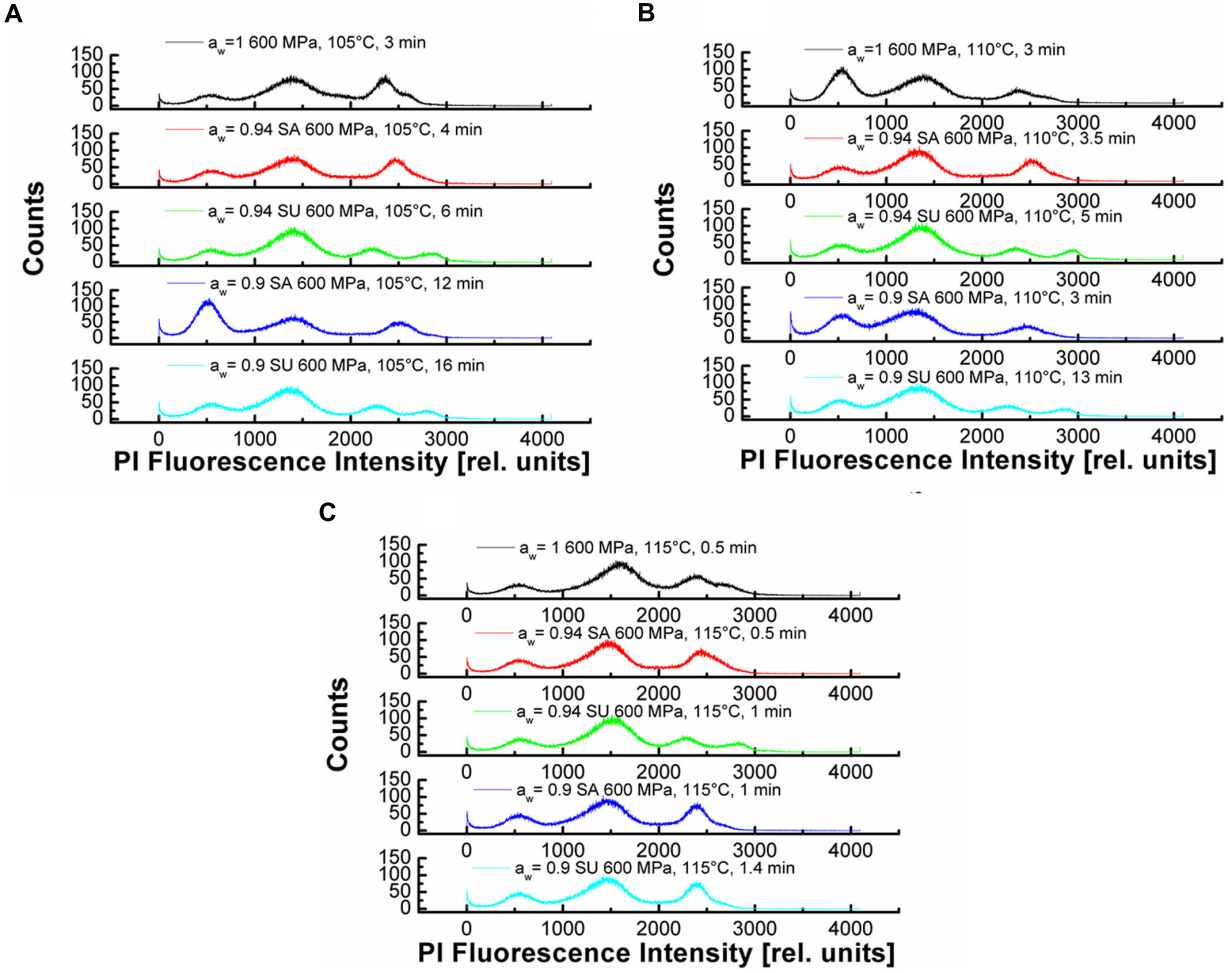
**Propidium iodide fluorescence intensity histogramms for different *a*_w_- values (SA = NaCl and SU = sucrose), temperatures and times. (A)** 105°C, 600 MPa with *a*_w_ = 1, 0.94, 0.90 for NaCl and sucrose **(B)** 110°C, 600 MPa with *a*_w_ = 1, 0.94, 0.90 for NaCl and sucrose **(C)** 115°C, 600 MPa with *a*_w_ = 1, 0.94, 0.90 for NaCl and sucrose.

Another way to illustrate the results of the FCM-analyses is by showing the histograms of the measurement. These results are depicted in dependencies of *a*_w_, temperature and time in **Figures 5A–C**. At 105°C, 600 MPa (**Figure 5A**) one can see that the PI Fluorescence Intensity (PIFI) is depending on the solute concentration, the *a*_w_ and the dwell time. The PIFI moves from high intensities at *a*_w_ = 1 down to lower overall PIFI for *a*_w_ = 0.9, although longer treatment times were applied for lower *a*_w_.

## Discussion

In the present paper the influence of two solutes (NaCl and sucrose) and their corresponding *a*_w_-values and their influence on inactivation of *B. amyloliquefaciens*, amount of DPA released and changes on the structural spore properties under HPTS conditions were studied.

High sucrose and high NaCl concentrations and corresponding *a*_w_ of ≤0.94 have a servere impact on the inactivation. As many researches have postulated and which was just recently proven by Olivier et al. (2015) that HP and high temperatures have synergistic effect on the spore inactivation. This effect could be used to treat spores already at lower temperatures and shorter dwell times to achieve similar inactivation as under thermal only conditions. Literature data on the influence of HP on spores suspended in different *a*_w_ solutions are rare. Nevertheless, these findings are in accordance to results obtained by Raso et al. (1998), who reported that the inactivation of *B. cereus* by HHP decreased when the *a*_w_ decreased with the addition of sucrose (non-ionic). These findings in this work and the findings of Raso et al. (1998) are in contradiction to Sale et al. (1970), who concluded that only ionic (NaCl etc.) solutes could protect the spores from being inactivated. The results here indicate that both solutes (NaCl and sucrose) both have a protective effect and the one of sucrose is enhanced. The results of Sale et al. (1970) were obtained in the same range of *a*_w_ as the ones described here but the applied kinetics was 100 MPa, 30 min at 65°C. Maybe in these temperature-pressure domain sucrose acts differently than at the conditions tested here or by Raso et al. (1998). Raso et al. (1998) tested also in the same *a*_w_ range but at pressures between 250 and 690 MPa at 40°C, so as mentioned before the differences could be caused by sucrose acting differently at higher pressures. Although, more research will be needed to prove this assumption. Further different sporulation conditions used in the papers could also have been the cause for the contradictory results.

The influence of the *a*_w_ adjusted by NaCl and sucrose on the DPA-release is given for *a*_w_ ≤ 0.94. If one looks exemplary on the behavior of the DPA-release in *a*_w_ = 0.92 by NaCl and sucrose, where 80–95% of DPA are released, in comparison to *a*_w_ = 1 in the temperature range 105–115°C one can see the influence quite nicely. At 105°C the time needed for 80–95% of DPA to be released is double (5 min for *a*_w_ = 0.92 NaCl) respectively 4 times (13 min for *a*_w_ = 0.92 sucrose) in comparison to *a*_w_ = 1 (3 min). This ratio does not change for an increase to 110°C but the times needed to achieve the same release at this temperature are of course lower. At 115°C only the sucrose keeps the ratio in comparison to the other samples.

With increasing temperature the baroprotective effects of the solutes are more and more suppressed. This leads at 115°C, 600 MPa (**Tables 1** and **2**) to similar released relative DPA amounts and affirms the assumption made under section “High Pressure High Temperature in Comparison to Thermal only Inactivation of *Bacillus amyloliquefaciens* in Different Water Activities” that the baroprotective effects of the solutes decreases with increasing temperature (Reineke et al., 2013b; Nagler et al., 2014). Therefore, the temperature plays a dominant role for the inactivation in the tested *a*_w_ as discussed by Reineke et al. (2013a,b) for buffer systems. Further, if one compares NaCl and sucrose at the same *a*_w_ (**Figure 4**) it shows that the ratio of relative DPA-release and maximal treatment time is lower for sucrose. This further indicates that the baroprotecive effect of sucrose on the HP high temperature inactivation of *B. amyloliquefaciens* is more pronounced than the one of NaCl. The proven and shown baroprotective effect of solutes on spores under HP high temperature conditions reduces and delays the release of DPA out of the spores if the *a*_w_ is ≤0.94 and therefore also the inactivation. For high NaCl concentrations (2.4–3.6 M) Nagler et al. (2014) stated that these concentrations can decelerate germination and decrease the overall germination efficiency under pressure. This might be due to an increase of the osmotic pressure in the outer and inner spore membrane and therefore lead to no water diffusion into the spore (Reineke, 2012; Nagler et al., 2014). This implies that a certain concentrations of sucrose and NaCl might be able to reinforce the ability of the spores to retain the DPA, but more research is needed to understand this mechanism of baroprotection completely. If this is due to the not completely functional DPA-protein channels, which might be in a dehydrated state; other spore compartments that are not correctly functional or their inner spore membrane is altered by the extrinsic factors. And/or DPA release is hindered due to the shortage of free water outside of the spore, which cannot be enlightened by HPLC alone. This hypothesis could be underlined by trials conducted at the Department of Food biotechnology and Process engineering of the Technische Universität Berlin (data not shown) with different water/oil concentrations. For 100 and 75% oil (olive and sunflower-oil) concentrations almost no inactivation occurred at 105°C 600 MPa (1 log_10_ inactivation took roundabout 30 min). With increasing water concentration 50 and 75% the inactivation rate increased drastically (-5.5 log_10_ inactivation in 3 respectively 7 min). These results support the hypothesis that free water must be available to guarantee a rapid inactivation and DPA-release.

The FCM-analysis is a sophisticated way to gain information on the physiological state of the spore and therefore gain more knowledge on the impact of HP high temperature processes on the inactivation. The method used by Mathys et al. (2007) is able to detected different kind of spore subpopulations (germinated, unknown state, inactivated) which stand for different membrane damage intensity and this can be used to identify possible influences of solutes on the spore membrane respectively inactivation under HP and high temperature conditions. This means that NaCl does have some kind of influence on the inner spore membrane. Although as shown for the inactivation data and the DPA-release the baroprotective effect is lost/is reduced at temperatures ≥115°C. For sucrose it seems like sucrose could have an influence on the dye since the concentrations of PI intensity are quite high, the values vary and the SD are high (**Figures 4D,E**). One is able to see similar tendencies as for NaCl but the influence of sucrose is not as distinct and dominant as seen for the inactivation kinetics and the DPA-release. As mentioned this could cohere with sucrose concentrations, which might interfere with the dye and therefore lead to an inconsistent staining.

This indicates that the baroprotective effect of the solutes is present for certain solute concentrations and that there is an influence of the solutes on the inner spore membrane. For the future the FCM analyses needs to be optimized to give consistent results in high concentrated solutions. Since the results obtained, at least for sucrose, leave a lot of room for interpretation. Further the *a*_w_ = 0.94 seems to be, as already described for the inactivation and the DPA-release, a threshold *a*_w_ where the baroprotective effect becomes pronounced. A similar trend is seen in **Figure 5B**, although here only *a*_w_ = 0.9 adjusted with sucrose shows a lower PIFI then the other samples. As mentioned, for the DPA-release and the inactivation, at 115°C, 600 MPa the PIFI shows no differences for the tested *a*_w_-values and further the dwell times applied are equal. At 115°C, 600 MPa the baroprotective effect of the solutes used to adjust the *a*_w_ is lost and the temperature plays the dominant role (**Figure 5C**).

## Conclusion

The aim of this work was to investigate the baroprotective influence of solutes (NaCl and sucrose) and the corresponding *a*_w_ (1-0.9) on the inactivation mechanisms of *B. amyloliquefaciens* in a temperature range of 105–115°C at 600 MPa. This work showed that for certain solute concentrations (corresponding to an *a*_w_ ≤ 0.94 and temperature ≤110°C) a baroprotective effect is present but a more rapid inactivation is possible if pressure and heat are applied together then only by heat. As other researchers already indicated the *a*_w_ respectively the substances responsible for the *a*_w_ can have an impact on the inactivation under HP high temperature conditions (Sevenich et al., 2013, 2014, 2015; Georget et al., 2015). Sucrose has a higher protective effect then NaCl but the effect is minimized when temperature ≥115°C at 600 MPa are used. The calculations of the isorate lines could be used to optimize HPTS processes in food systems where NaCl and sucrose are the major solutes/ingredients. Based on this a 12 D-concept could be established as an orientation for more complex foods with similar *a*_w_ values, such as liquid foods.

The DPA-release is slowed down by lower *a*_w_ values which might be due to interactions of the solutes with the inner membrane as the results of the FCM analyses indicate. More research is needed in the future to fully understand these effects. This work was able to point out that solutes have an impact on the spore inactivation under HP. Although the influence at 600 MPa on a retarded inactivation is depending on the concentration, the solute and the temperature applied. As described for spore inactivation in aqueous solutions (*a*_w_ 1) the release of DPA is crucial for spore inactivation under pressure and the inner spore membrane is the presumably the target structure affected by HPs, high temperatures and by solutes, as shown in this study. For the future, to further look into the physiological changes in the spores due to solute uptake, possible tools could be the use of transmission electron microscopy (TEM) analyses.

Furthermore, the mechanisms of individual solutes and food matrices need to be fully understood in order to optimize the process design of HPTS. Therefore it is of great importance to analyze at what time and at what pressure/temperature combinations the baroprotective effect of typical food ingredients occur and in what way do these affect spore components. HP processing proved valuable to overcome protective effects of solutes and achieve shorter process times for sterilization under HP. The gained data could be used as a basis for the inactivation behavior of spores in real food systems under the same *a*_w_-value conditions and lead to case by case optimized treatment times for foods containing mainly sucrose or salt.

## Conflict of Interest Statement

The authors declare that the research was conducted in the absence of any commercial or financial relationships that could be construed as a potential conflict of interest.
